# Differential Metabolites in Chinese Autistic Children: A Multi-Center Study Based on Urinary ^1^H-NMR Metabolomics Analysis

**DOI:** 10.3389/fpsyt.2021.624767

**Published:** 2021-05-11

**Authors:** Yu Ma, Hao Zhou, Chunpei Li, Xiaobing Zou, Xuerong Luo, Lijie Wu, Tingyu Li, Xiang Chen, Meng Mao, Yi Huang, Erzhen Li, Yanpeng An, Lili Zhang, Tianqi Wang, Xiu Xu, Weili Yan, Yonghui Jiang, Yi Wang

**Affiliations:** ^1^Department of Neurology, Children's Hospital of Fudan University, Shanghai, China; ^2^Department of Pediatrics, Guizhou Provincial People's Hospital, Guiyang, China; ^3^Child Development Behaviour Centre, The Third Affiliated Hospital, Sun Yat-sen University, Guangzhou, China; ^4^Department of Psychiatry, The Second Xiangya Hospital of Central South University, Changsha, China; ^5^Department of Children and Adolescent Health, School of Public Health, Harbin Medical University, Harbin, China; ^6^Department of Child Health Care, Children's Hospital of Chongqing Medical University, Chongqing, China; ^7^The Second Affiliated Hospital and Yuying Children's Hospital of Wenzhou Medical University, Wenzhou, China; ^8^Department of Child Health Care, Chengdu Women and Children's Hospital, Chengdu, China; ^9^Department of Psychiatry, West China Hospital of Sichuan University, Chengdu, China; ^10^Department of Neurology, Capital Institute of Paediatrics, Beijing, China; ^11^State Key Laboratory of Genetic Engineering, Metabonomics and Systems Biology Laboratory, School of Life Sciences, Fudan University, Shanghai, China; ^12^Department of Child Health Care, Children's Hospital of Fudan University, Shanghai, China; ^13^Department of Clinical Epidemiology, Children's Hospital of Fudan University, Shanghai, China; ^14^Department of Genetics and Paediatrics, Yale School of Medicine, New Haven, CT, United States

**Keywords:** autism spectrum disorder, ^1^H-NMR analysis, metabolomics, urine, amino acid metabolite

## Abstract

**Background:** Autism spectrum disorder (ASD) is a group of early-onset neurodevelopmental disorders. However, there is no valuable biomarker for the early diagnosis of ASD. Our large-scale and multi-center study aims to identify metabolic variations between ASD and healthy children and to investigate differential metabolites and associated pathogenic mechanisms.

**Methods:** One hundred and seventeen autistic children and 119 healthy children were recruited from research centers of 7 cities. Urine samples were assayed by ^1^H-NMR metabolomics analysis to detect metabolic variations. Multivariate statistical analysis, including principal component analysis (PCA), and orthogonal projection to latent structure discriminant analysis (OPLS-DA), as well as univariate analysis were used to assess differential metabolites between the ASD and control groups. The differential metabolites were further analyzed by receiver operating characteristics (ROC) curve analysis and metabolic pathways analysis.

**Results:** Compared with the control group, the ASD group showed higher levels of glycine, guanidinoacetic acid, creatine, hydroxyphenylacetylglycine, phenylacetylglycine, and formate and lower levels of 3-aminoisobutanoic acid, alanine, taurine, creatinine, hypoxanthine, and N-methylnicotinamide. ROC curve showed relatively significant diagnostic values for hypoxanthine [area under the curve (AUC) = 0.657, 95% CI 0.588 to 0.726], creatinine (AUC = 0.639, 95% CI 0.569 to 0.709), creatine (AUC = 0.623, 95% CI 0.552 to 0.694), N-methylnicotinamide (AUC = 0.595, 95% CI 0.523 to 0.668), and guanidinoacetic acid (AUC = 0.574, 95% CI 0.501 to 0.647) in the ASD group. Combining the metabolites creatine, creatinine and hypoxanthine, the AUC of the ROC curve reached 0.720 (95% CI 0.659 to 0.777). Significantly altered metabolite pathways associated with differential metabolites were glycine, serine and threonine metabolism, arginine and proline metabolism, and taurine and hypotaurine metabolism.

**Conclusions:** Urinary amino acid metabolites were significantly altered in children with ASD. Amino acid metabolic pathways might play important roles in the pathogenic mechanisms of ASD.

## Introduction

Autism spectrum disorder (ASD) is a group of early-onset neurodevelopmental disorders characterized by social communication difficulties, narrow interests, and repetitive stereotyped behaviors (1). In the United States, the prevalence of ASD has increased in recent years, ranging from 1.57% in 2009 to 1.85% in 2020 (2, 3). The prevalence of ASD among children aged 6–12 years is ~0.70% in China (4). The large cost associated, mainly consisting of special education services and parental productivity loss, has caused a heavy burden to society and families (5). Despite the lack of effective drug treatments, several studies highlight the potential benefits of early diagnosis and parent-mediated interventions, which have to some extent improved children's social and communicative abilities (6, 7). The detection rate of genetic etiology of ASD is about 10–15% (8–10). However, early diagnosis remains a challenge for non-genetic ASD, which is mainly based on combining clinician observation with caregiver reports (11). Currently, there is an urgent need to find valuable biomarkers for the early diagnosis of ASD.

Some metabolomic studies have indicated the presence of elevated biomarkers in blood and urine samples from ASD patients, and these biomarkers include pyruvate, lactate, and mitochondrial-related enzymes (12–14). Major analytical techniques for metabolomics are nuclear magnetic resonance spectroscopy (NMR) and chromatography, including gas chromatography–mass spectrometry (GC-MS) and liquid chromatography–mass spectrometry (LC-MS). The advantages of ^1^H-NMR are that the sample preprocessing is simple and non-destructive and that the detection of metabolites is comprehensive (15, 16). In 2010, Yap et al. first used urinary ^1^H-NMR analysis to detect potential biomarkers for ASD (17). Over the last few years, with urinary ^1^H-NMR analysis, some discriminating metabolites have been identified between ASD and healthy people. Studies indicated that ASD group showed higher levels of hippurate, glycine, tryptophan and D-threitol and lower levels of glutamate, creatine, valine, betaine and 3-methylhistidine. Further analyses indicated possible pathogenic mechanisms involving gut microbial metabolism, oxidative stress conditions and amino acid metabolism (18, 19). Overall, ^1^H-NMR analysis shows great potential for the identification of biochemical signatures and investigation of the disease mechanisms of ASD. However, previous ^1^H-NMR analyses lack large-scale sample sizes to confirm the significance and connection between metabolites and ASD. We aimed to conduct a large-scale and multi-center study to identify metabolic variations between ASD and control groups through urinary ^1^H-NMR metabolomics analysis and to investigate potential biological mechanisms related to differential metabolites.

## Methods

### Participants

The study was conducted from January 2014 to December 2016. All the participants were recruited from research centers of 7 cities (Shanghai, Guangzhou, Changsha, Chongqing, Chengdu, Wenzhou, and Beijing). Participants were drawn from ASD and control group.

Autistic children from both hospitals and local autism rehabilitation of each research center were enrolled in the ASD group. The inclusion criteria for ASD group were: (a) children aged 2–18 years; (b) no limitation on the gender; (c) the diagnosis of ASD was based on the Diagnostic and Statistical Manual of Mental Disorders 5 (DSM-5) criteria (1) and confirmed with the Autism Diagnostic Observation Schedule (ADOS) and the Autism Diagnostic Interview-Revised (ADI-R) criteria by trained clinical psychiatrists from each research center; (d) urine sample was available. Exclusion criteria for ASD group were: (a) symptomatic autism (such as Rett syndrome and fragile X syndrome); (b) other mental illness (such as attention-deficit hyperactivity disorder); (c) other neurological disorders (such as epilepsy and central nervous system infections); (d) inherited metabolic diseases; (e) history of brain injury; (f) taking non-essential drug or dietary supplement before (72 h) and during sample collection.

Healthy children from the health examination center of each research center were enrolled in the control group. The inclusion criteria for control group were: (a) children with no abnormality in health examination and typical development; (b) age- and sex-matching with ASD group; (c) urine sample was available. Exclusion criteria for control group were: (a) siblings of ASD group; (b) clinical evidence of ASD diagnosis; (c) mental illness (such as attention-deficit hyperactivity disorder); (d) neurological disorders (such as epilepsy and central nervous system infections); (e) inherited metabolic diseases; (f) history of brain injury; (g) taking non-essential drug or dietary supplement before (72 h) and during sample collection.

After all eligible participants and their parents provided informed consent, they were invited to participate in the study. The study was approved by the institutional ethics committee at the Children's Hospital of Fudan University.

### Sample Collection

First morning urine specimens were collected from all participants during the research period. Samples were collected in 15 mL urine collection tubes without preservative. Each sample was centrifuged and aliquoted into 1.5 mL EP tubes. Afterwards, samples were numbered (“1” represents the ASD group, and “2” represents the control group) and stored at −80°C immediately until ^1^H-NMR analysis.

### ^1^H-NMR Spectroscopy Experiments and Data Processing

#### Sample Preparation

A 500 μL urine sample was added to a 1.5 mL EP tube prefilled with 14 μL KF (5 M) solution. After vortexing, the sample was allowed to rest for 10 min, followed by centrifugation (12,000 rpm, 4°C) for 10 min. A total of 450 μL liquid supernatant was added to an NMR tube preloaded with 10 μL EDTA-d12 (0.1 M). The NMR tube cap was covered and mixed by hand. Finally, 45 μL Na^+^/K^+^ buffer (1.5 M, pH = 7.40) was added to the NMR tube, which was mixed by hand and then placed in the NMR spectrometer for data collection. The sample preparation process for ^1^H-NMR analysis is shown in Supplementary Figure 1.

#### ^1^H-NMR Spectroscopy Experiments

All ^1^H-NMR spectroscopy experiments were performed at 298 K using a Bruker AVIII 600 MHz NMR spectrometer (Bruker BioSpin, Germany) with a proton resonance frequency of 600.13 MHz.

The NOESYGPPR1D pulse sequence (RD-90°-t1-90°-tm-90°-ACQ) was used to collect the spectra. The parameters were set as follows: spectral width (SW) was 20 ppm, recycle delay (RD) was 2 s, mixing time (t_m_) was 80 ms, t_1_ was 4 μs, 90° pulse length was 14.8 s, data time was 1.36 s, data points were 32 K, and free induction decay (FID) accumulation was 64 times.

#### Data Processing

Spectra were processed using MestReNova software (MestReNova 8.1, Spain). The ^1^H-NMR FID signals were multiplied by an exponential function equivalent to a line broadening of 1 Hz before performing an automatic Fourier transformation. The phase distortion and baseline of each spectrum were manually adjusted. The internal standard trimethylsilyl propanoic acid (TSP, δ = 0 ppm) was used as the baseline to calibrate the chemical shifts. The concentration of TSP was 0.261 mmol/L, and the spectrum of TSP represented 12 ^1^H. The regions of the ^1^H-NMR spectra (δ 0.3–9.5 ppm) were divided into consecutive integrated spectral regions of equal width (δ 0.004 ppm). The spectral region of the water (δ 4.71–5.055 ppm) and urea (δ 5.6–6.12 ppm) peaks were removed from each spectrum to minimize variations caused by the presaturation of the residual water and urea resonances. Mnova software was used to correct the spectra with obvious chemical shifts after the integration. Metabolites were assigned by referencing the values for chemical shifts in J-resolved (JRES), COZY, TOCSY, HSQC, and HMBC spectra and literature reports. A series of 2D NMR spectra were acquired for selected samples.

To eliminate the instrument differences of sensitivity and stability and to reduce the analysis errors caused by the concentration differences of the samples, two normalization methods were performed. Creatinine normalization: the ^1^H-NMR spectra were normalized by using the creatinine methylene resonance (δ = 4.05 ppm) as a reference. Total area normalization (20): the integrated area in each bucket was normalized by the total sum of peak intensities to eliminate the effects of variable concentration among different samples.

### Data and Statistical Analysis

#### Clinical Characteristics of Participants

Difference in age between the ASD and control groups was evaluated by Student's *t-*test when the distribution was normal or the Mann-Whitney U test when it was skewed. Difference in sex was investigated using chi-square test. Statistical analyses were performed by using the SPSS statistical package program (version 20, SPSS Inc., Chicago, IL, USA), and *P* < 0.05 was considered statistically significant.

#### Multivariate Analysis

The normalized data were imported into the SIMCA-P+ software package (version 13.0, Umetrics, Sweden) for multivariate statistical analysis, including principal component analysis (PCA) and orthogonal projection to latent structure discriminant analysis (OPLS-DA) (21–24). PCA was first used to observe the overall distribution among samples and the stability of the whole analysis process. Abnormal data, which related to sample contamination or improper sampling, were removed according to the overall aggregation trend in all the samples. After which OPLS-DA was used to distinguish the overall difference in the metabolic profile and to find differential metabolites between the groups.

To prevent model overfitting, an internal validation method was used to verify the validity of the model. The OPLS-DA model was validated by a 7-fold cross-validation (CV) (25). R^2^ and Q^2^ were two parameters to assess the quality of the model. OPLS-DA was further validated by variance analysis of the cross-validated residuals (CV-ANOVA) (26), and the model was considered valid at *P* < 0.05.

After multiplying the loading value of each variable with its standard deviation, backtracking conversion of the data was performed. Then, the data were assessed by multivariate analysis and imported into mapping software based on MATLAB (version 7.1, USA) to plot the loading diagram of the correlation coefficient. The Pearson correlation coefficient represented the linear correlation between the variable and the first principal component score value of the OPLS-DA model and was used to determine whether the variation contributed significantly to the intergroup differentiation. The significance was evaluated by the threshold value of the absolute correlation coefficient, which was determined according to the confidence interval of the sample size. If the absolute value of the Pearson correlation coefficient of the variable was higher than the threshold value (*P* < 0.05), the content of the variable was considered significantly different between groups.

#### Univariate Analysis

Differential metabolites between the two groups were selected for univariate analysis. Data from the total area normalized peak area are expressed as the mean ± SD. An independent sample *t-*test was used for comparing the two groups when the distribution was normal. The non-normal distribution data were evaluated by Mann-Whitney U test. *P* < 0.05 was considered statistically significant. The false discovery rate (FDR) was used to correct multiple hypothesis testing. FDR *P* < 0.1 was considered statistically significant. We applied correlation analysis to detect the relationship between differential metabolite levels and age.

#### Receiver Operating Characteristics (ROC) Curve Analysis

The sensitivity and specificity of metabolites with significant differences between the two groups in the diagnosis of ASD were evaluated using a ROC curve analysis. ROC curves were generated by using MedCalc statistical software (version 19.1.7, Belgium). The area under the curve (AUC) was used to measure the overall degree of identification power. An AUC > 0.7 was considered acceptable. Optimal cut-off points were determined by maximizing the Youden's J index (J = sensitivity + specificity – 1). The sensitivity, specificity, false-negative rate (FNR), false-positive rate (FPR), positive predictive value (PPV), negative predictive value (NPV), likelihood ratio positive (LR+), and likelihood ratio negative (LR–) were calculated to compare the diagnostic accuracy of the metabolites. Further analysis was performed after each group was stratified by sex and age. Logistic regression was used to analyse the combined metabolites.

#### Metabolic Pathways and Network Analysis

MetaboAnalyst (version 4.0, https://www.metaboanalyst.ca/) was used for pathway and network analyses (27). All differential metabolites were imported into the pathway analysis module to obtain matched pathways according to the *P*-values from the pathway enrichment analysis and pathway impact values from the pathway topology analysis. Significantly affected metabolic pathways associated with differential metabolites were identified by metabolite set enrichment analysis (MSEA). A metabolite-gene-disease interaction network was established to detect the connections of differential metabolites and associated pathways.

## Results

### Characteristics of the ASD and Control Groups

A total of 117 children with ASD were enrolled in the ASD group, and 119 healthy children were enrolled in the control group. The mean ages of the ASD and control groups were 10.12 ± 2.60 and 9.90 ± 1.73, respectively, with no significant difference between them (*P* = 0.468). The male to female ratios were 4.82:1 in the ASD group and 3.58:1 in the control group, with no significant difference between them (*P* = 0.388).

### ^1^H-NMR Spectrum of Urine Samples

**Figure 1:**
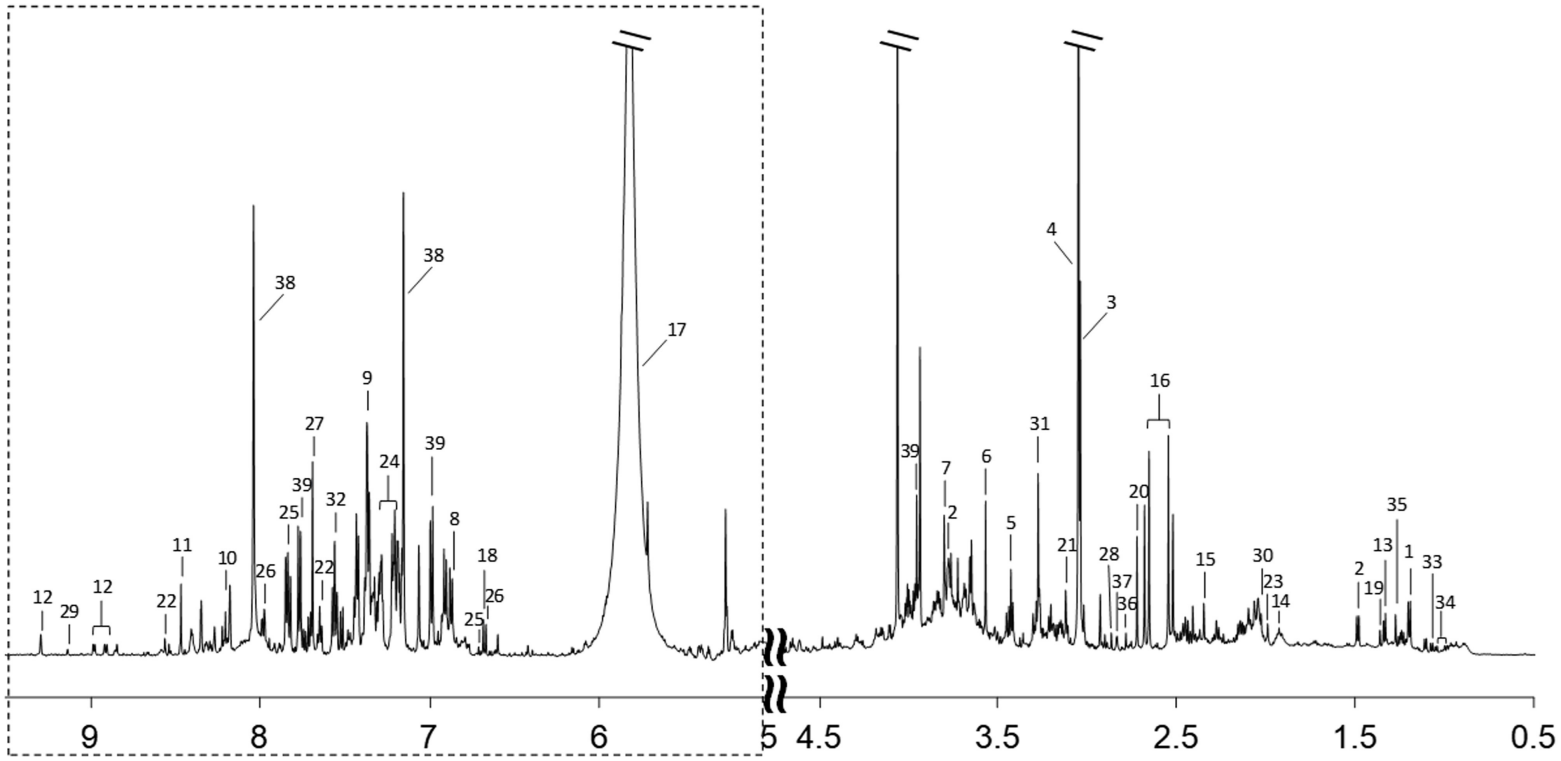
A typical 600 MHz ^1^H-NMR spectrum of a urine sample. Region of dashed box is vertically magnified 8 times. Metabolites corresponding to each number are listed in Supplementary Table 1.

^1^H-NMR spectra of urine samples from all participants were collected. A typical ^1^H-NMR spectrum is shown in Figure 1. The keys for metabolites in Figure 1 are given in Supplementary Table 1. A total of 39 metabolites were identified in the ^1^H NMR spectra of urine samples.

### Multivariate Analysis of ^1^H-NMR Spectra of Urine Samples

#### Creatinine Normalization Analysis

**Figure 2:**
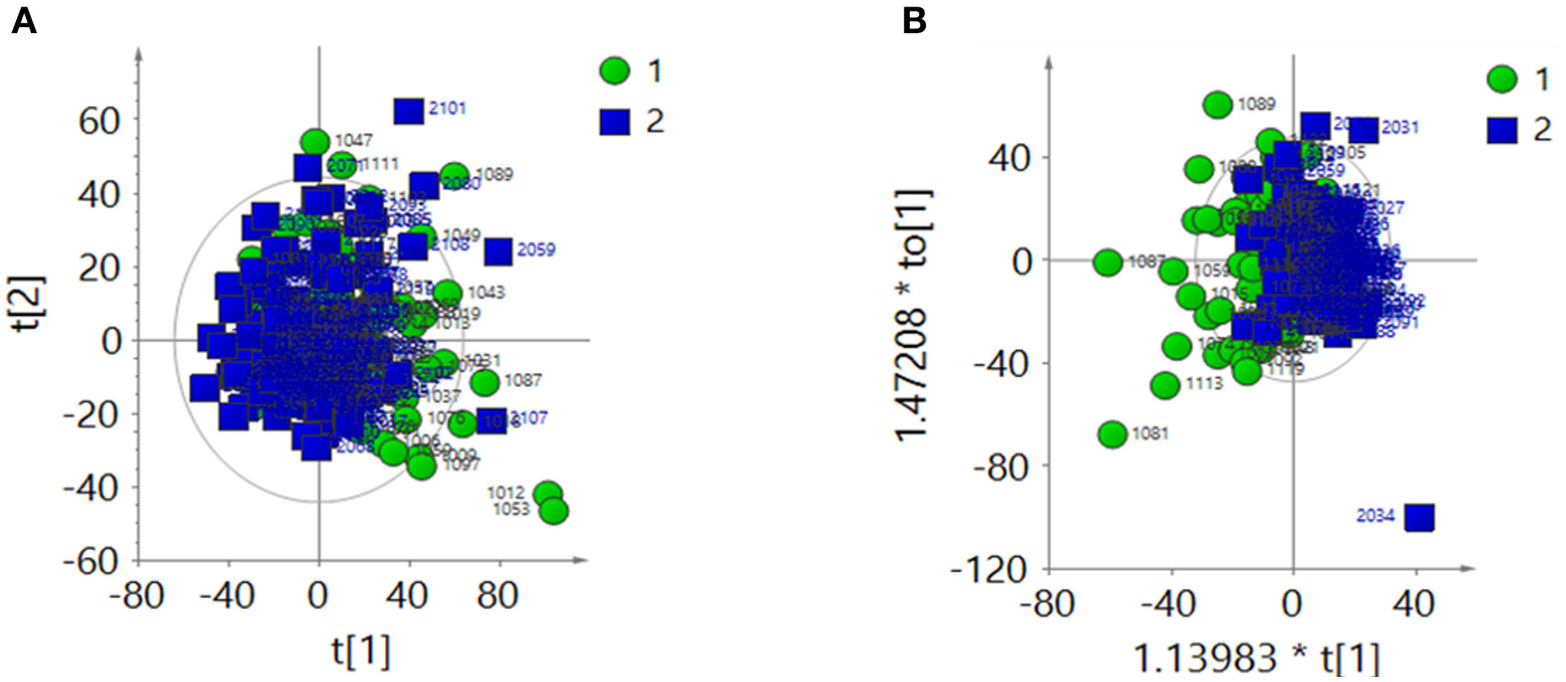
After the creatinine normalization, multivariate analysis results of ^1^H-NMR spectra of urine samples from the ASD group (green dots, 1) and control group (blue squares, 2). **(A)** PCA score plot: R^2^X = 0 403, Q^2^ = 0.135. **(B)** OPLS-DA score plot: R^2^X = 0.257, Q^2^ = 0.0138, CV-ANOVA *P* = 0.523.

After creatinine normalization, PCA was performed on ^1^H-NMR spectra of urine samples. Discriminant variables obtained from the PCA score plot (Figure 2A) were R^2^X = 0.403 and Q^2^ = 0.135. The OPLS-DA score plot (Figure 2B) showed R^2^X = 0.257 and Q^2^ = 0.0138. CV-ANOVA of the OPLS-DA model indicated that the model was not valid (*P* = 0.523).

#### Total Area Normalization Analysis

**Figure 3:**
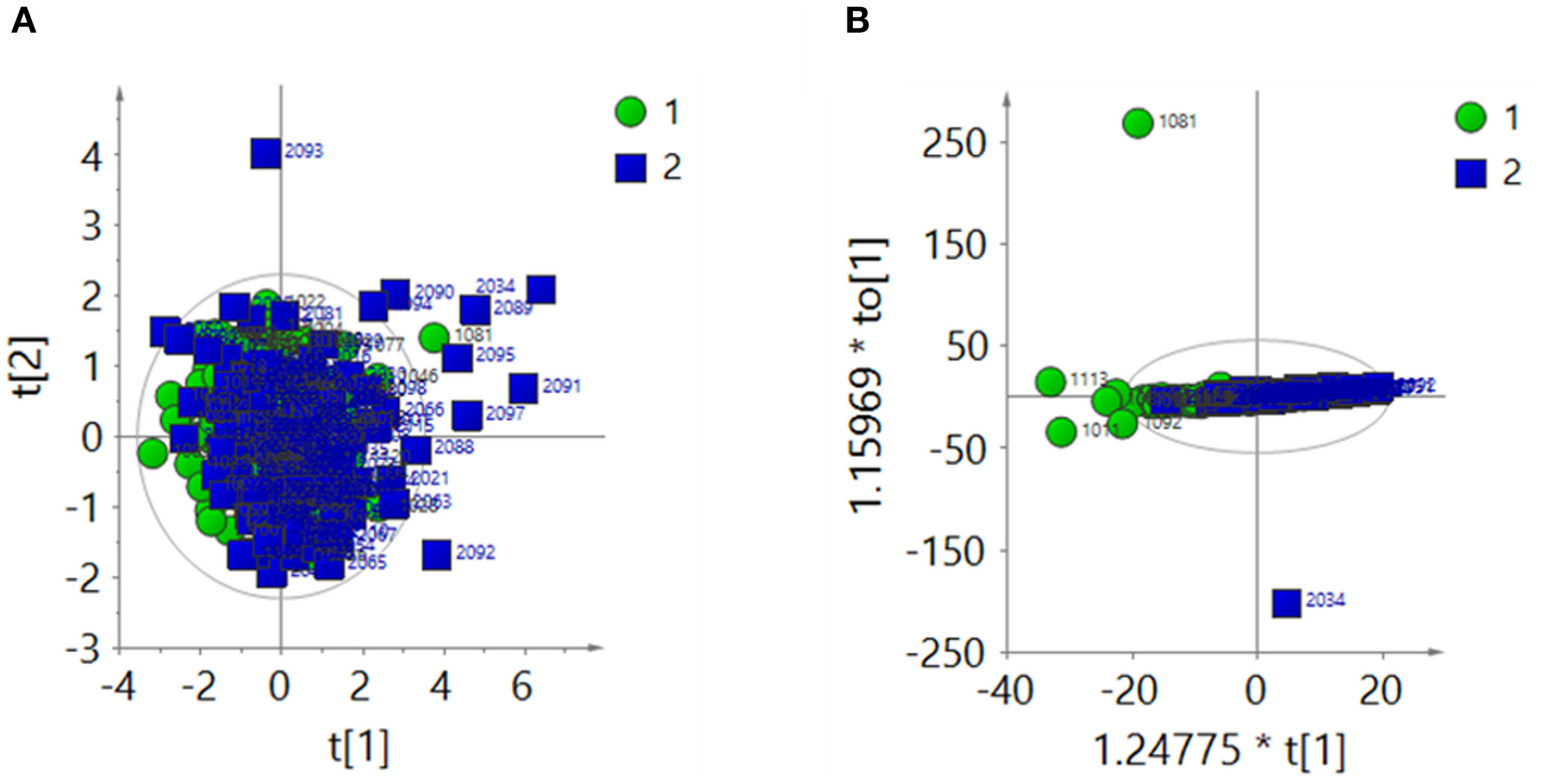
After the total area normalization, multivariate analysis results of ^1^H-NMR spectra of urine samples from the ASD group (green dots, 1) and control group (blue squares, 2). **(A)** PCA score plot: R^2^X = 0.366, Q^2^ = 0.0656. **(B)** OPLS-DA score plot: R^2^X = 0.274, Q^2^ = 0.0565, CV-ANOVA *P* = 0.009.

The ^1^H-NMR spectra of the two groups were analyzed by PCA after total area normalization. The parameters of the PCA score plot (Figure 3A) were R^2^X = 0.366 and Q^2^ = 0.0656. The OPLS-DA score plot (Figure 3B) showed R^2^X = 0.274, Q^2^ = 0.0565 and CV-ANOVA *P* = 0.009. These results suggested that the model was valid.

### Differential Metabolites Between the ASD and Control Groups

**Figure 4:**
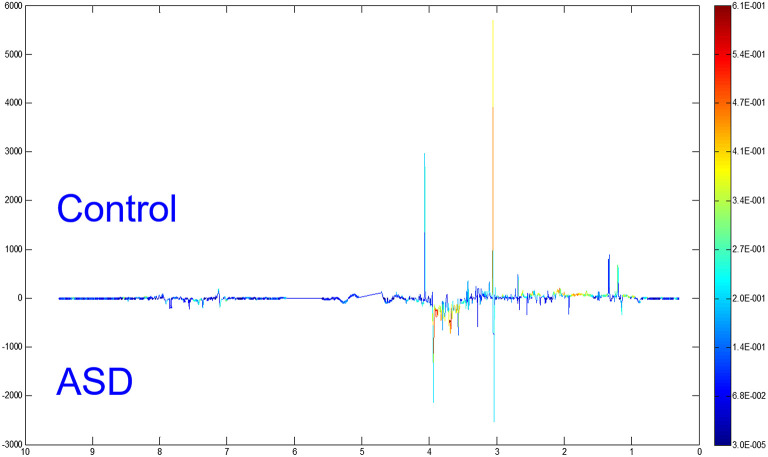
OPLS-DA model: a polychromatic correlation coefficient loading plot.

To distinguish the metabolic differences between the two groups, a polychromatic correlation coefficient loading plot (Figure 4) was drawn. The color of the polychromatic loading plot was encoded by the absolute value of the correlation coefficient. The warmer the color is, the higher the absolute value of the correlation coefficient and the greater the contribution to the intergroup differentiation. The threshold of the absolute value of the Pearson's correlation coefficient was determined to be 0.182. The variables corresponding to the correlation coefficient with an absolute value > 0.182 contributed significantly to the intergroup differentiation (*P* < 0.05). Differential metabolites identified by the Pearson's correlation coefficient were showed in Table 1. Compared with the control group, the ASD group showed higher levels of glycine, guanidinoacetic acid, creatine, hydroxyphenylacetylglycine, phenylacetylglycine and formate and lower levels of 3-aminoisobutanoic acid, alanine, taurine, creatinine, hypoxanthine and N-methylnicotinamide.

**Table 1:** Pearson's correlation coefficient of discriminant metabolites.

**Metabolites**	Pearson's correlation coefficient^*^
3-Aminoisobutanoic acid	0.208
Alanine	0.220
Taurine	0.242
Glycine	−0.237
Guanidinoacetic acid	−0.424
Creatine	−0.278
Creatinine	0.268
Hydroxyphenylacetylglycine	−0.190
Phenylacetylglycine	−0.233
Hypoxanthine	0.370
Formate	−0.248
N-methylnicotinamide	0.348
Unknown	−0.296

* *The negative value indicates that the metabolite content in the urine samples of the control group is significantly lower than that of the ASD group. Conversely, the positive value indicates that the metabolite of the control group is significantly higher than that of the ASD group*.

**Figure 5:**
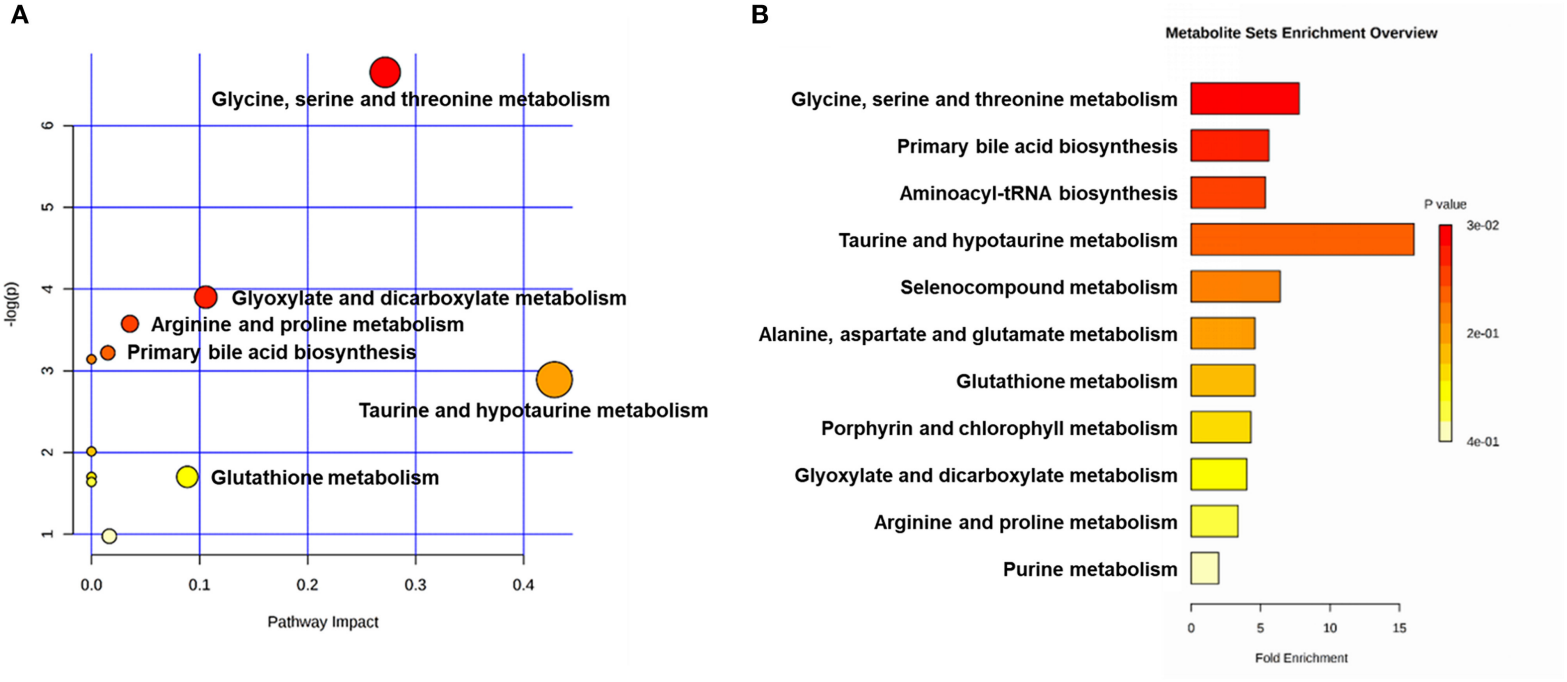
Metabolic pathway analyses utilizing the MetaboAnalyst functional interpretation tools. **(A)** Bubble plot of metabolic pathway impact. The metabolic pathways are shown as bubbles. The X coordinate and size of the bubble represent the value of pathway impact in the topology analysis. The Y coordinate and color of the bubble represent the *P-*value of the enrichment analysis. The darker red color and larger size indicate a more significant metabolite change in the corresponding pathway. **(B)** Metabolite set enrichment analysis (MSEA) plot. Significantly enriched pathways are represented by bars. The color and length of the bar are based on the *P-*value and fold enrichment, respectively.

The fold changes of differential metabolites are summarized in Figure 5. A total of 6 metabolites, including 3-aminoisobutanoic acid (*P* = 0.0425), creatine (*P* = 0.0009), creatinine (*P* < 0.0001), hypoxanthine (*P* < 0.0001), formate (*P* = 0.0267), and N-methylnicotinamide (*P* = 0.0149), showed significant differences in the normalized peak areas between the two groups. The differences of above 6 metabolites remained significant after correction for multiple hypothesis testing (FDR *P* < 0.1) (Table 2). Correlation analysis showed that the level of creatinine was positively correlated with age in the ASD group (r = 0.215, 95% CI 0.013 to 0.401, *P* = 0.0370). In the control group, the level of creatinine was also positively correlated with age (r = 0.215, 95% CI 0.036 to 0.380, *P* = 0.0190), and creatine was negatively correlated with age (r = −0.277, 95% CI −0.435 to −0.102, *P* = 0.0023).

**Table 2:** Peak areas of differential metabolites after the total area normalization.

**Metabolites**	**ASD group**		**Control group**		*P*-value^*^	FDR *P*-value^*^	**Fold change**
	**Mean**	**SD**	**Mean**	**SD**			
3-Aminoisobutanoic acid	0.77	0.61	0.94	0.68	**0.0425**	**0.0921**	0.82
Alanine	0.52	0.16	0.51	0.17	0.8440	0.9975	1.01
Taurine	0.63	0.21	0.62	0.17	0.9162	0.9162	1.00
Glycine	1.24	0.39	1.19	0.44	0.3430	0.4459	1.04
Guanidinoacetic acid	1.42	0.32	1.36	0.36	0.1446	0.2350	1.05
Creatine	1.78	0.86	1.43	0.72	**0.0009**	**0.0039**	1.24
Creatinine	4.68	0.90	5.26	1.21	**<0.0001**	**0.0012**	0.89
Hydroxyphenylacetylglycine	0.15	0.06	0.15	0.06	0.8860	0.9598	1.01
Phenylacetylglycine	0.48	0.20	0.44	0.21	0.1097	0.2037	1.10
Hypoxanthine	0.02	0.01	0.04	0.03	**<0.0001**	**0.0007**	0.62
Formate	0.06	0.04	0.05	0.02	**0.0267**	**0.0694**	1.17
N-methylnicotinamide	0.01	0.01	0.01	0.01	**0.0149**	**0.0484**	0.79
Unknown	0.26	0.34	0.22	0.18	0.3109	0.4491	1.16

* *Bold value indicate that the difference was considered statistically significant (P < 0.05, FDR P < 0.1)*.

### Differential Metabolites and Potential Biological Mechanisms

#### The Sensitivity and Specificity of Metabolites in the Diagnosis of ASD

The diagnostic accuracies of differential metabolites in the two groups were evaluated by ROC curve analysis (Table 3, Supplementary Figure 2). The ROC curve showed relatively significant diagnostic values of hypoxanthine (AUC = 0.657, 95% CI 0.588 to 0.726), creatinine (AUC = 0.639, 95% CI 0.569 to 0.709), creatine (AUC = 0.623, 95% CI 0.552 to 0.694), N-methylnicotinamide (AUC = 0.595, 95% CI 0.523 to 0.668) and guanidinoacetic acid (AUC = 0.574, 95% CI 0.501 to 0.647) for ASD (Supplementary Figure 3). The AUC of ROC analysis of the creatine/creatinine ratio was 0.6480 (95% CI 0.579 to 0.718). For each metabolite, there was no significant difference in AUCs between males and females (Supplementary Table 2). Compared with age stratification of 7–9 years old, the metabolites guanidinoacetic acid and creatine showed significantly higher diagnostic accuracy for ASD in the age stratification of 13–15 years old (AUC of guanidinoacetic acid = 0.802, 95% CI 0.566 to 0.944, *P* = 0.0282; AUC of creatine = 0.823, 95% CI 0.589 to 0.955, *P* = 0.0344. Results shown in Supplementary Table 3). By combining the metabolites of creatine, creatinine and hypoxanthine, the AUC of the ROC curve reached 0.720 (95% CI 0.659 to 0.777), with a sensitivity of 80.34% and specificity of 52.94%.

**Table 3:** Diagnostic accuracies of differential metabolites between the ASD and control groups.

**Metabolites**	**AUC**	**Cut-off**	**Sensitivity (%)**	**Specificity (%)**	**FNR (%)**	**FPR (%)**	**PPV (%)**	**NPV (%)**	**LR+**	**LR–**
3-Aminoisobutanoic acid	0.568	0.5890	67.52	50.42	32.48	49.58	57.2	61.2	1.36	0.64
Alanine	0.508	0.4190	73.50	31.93	26.50	68.07	51.5	55.1	1.08	0.83
Taurine	0.502	0.5683	44.44	61.34	55.56	38.66	53.1	52.9	1.15	0.91
Glycine	0.560	1.2698	41.88	70.59	58.12	29.41	58.3	55.3	1.42	0.82
Guanidinoacetic acid	0.574	1.2573	76.07	40.34	23.93	59.66	55.6	63.2	1.27	0.59
Creatine	0.623	1.8805	44.44	77.31	55.56	22.69	65.8	58.6	1.96	0.72
Creatinine	0.639	4.6505	55.56	69.75	44.44	30.25	64.4	61.5	1.84	0.64
Hydroxyphenylacetylglycine	0.504	0.1710	26.50	78.99	73.50	21.01	55.4	52.2	1.26	0.93
Phenylacetylglycine	0.571	0.2742	87.18	26.89	12.82	73.11	54.0	68.1	1.19	0.48
Hypoxanthine	0.657	0.0359	88.03	36.13	11.97	63.87	57.5	75.4	1.38	0.33
Formate	0.529	0.0768	19.66	91.60	80.34	8.40	69.7	53.7	2.34	0.88
N-methylnicotinamide	0.595	0.0104	74.36	49.58	25.64	50.42	59.2	66.3	1.47	0.52

*AUC, area under the curve; FNR, false-negative rate; FPR, false-positive rate; PPV, positive predictive value; NPV, negative predictive value; LR+, likelihood ratio positive; and LR–, likelihood ratio negative*.

#### Correlated Metabolic Pathways and Networks

**Figure 6:**
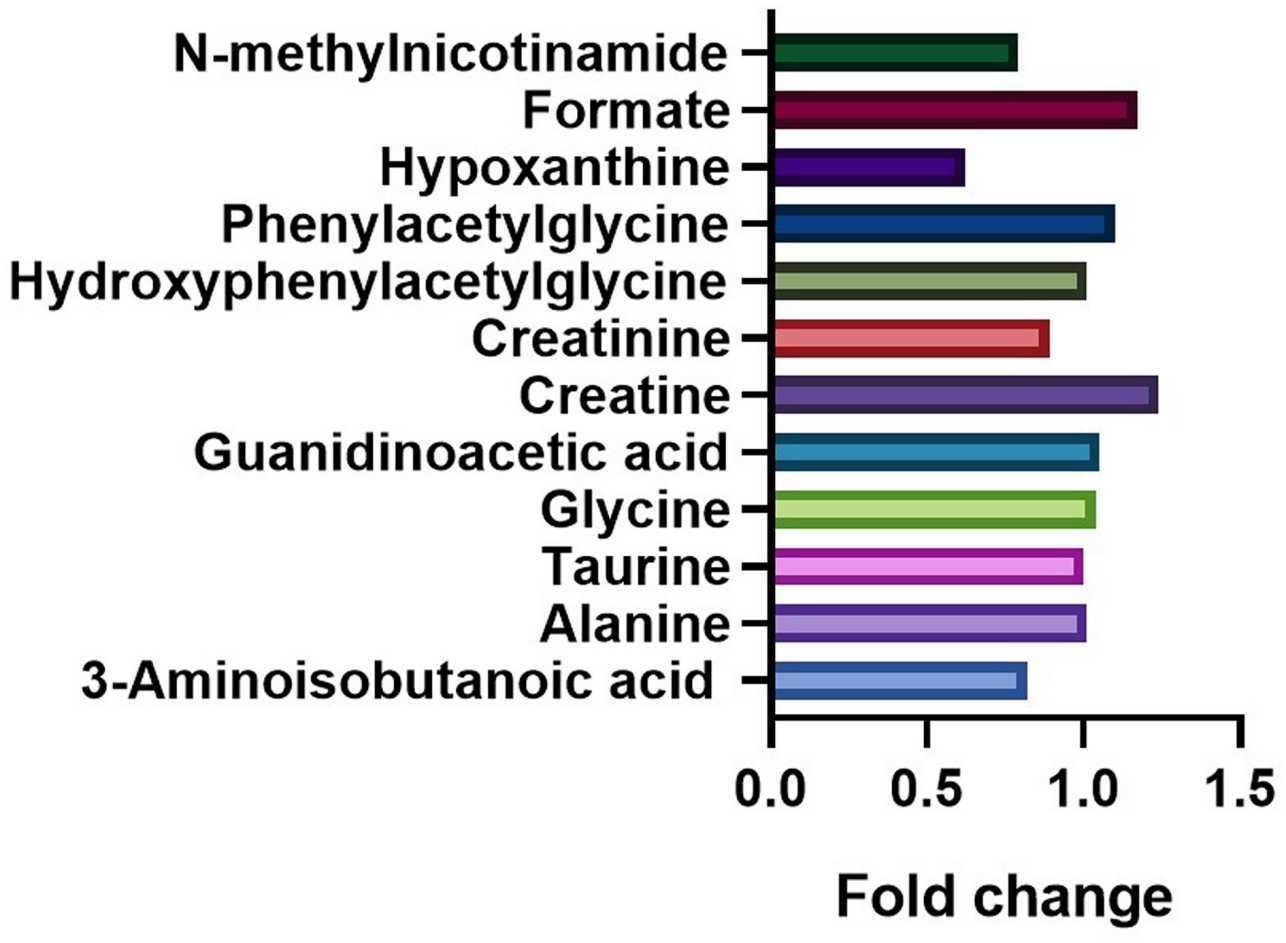
Comparation of the fold changes of the differential metabolites between the ASD and control groups. The fold change represents the ratio of average peak area between ASD group and control group.

The main metabolic pathways associated with differential metabolites are shown in Supplementary Figure 4. According to the bubble plot of the metabolic pathway impact, there were significant metabolite changes in the glycine, serine and threonine metabolism, glyoxylate and dicarboxylate metabolism and taurine and hypotaurine metabolism pathways (Figure 6A). The plot of metabolite set enrichment analysis (MSEA) listed the significant enrichment pathways of differential metabolites (Figure 6B). Glycine, serine and threonine metabolism, primary bile acid biosynthesis and aminoacyl-tRNA biosynthesis were the pathways with the most significant enrichment. The metabolite-gene-disease interaction network provides a global view of the connection of the differential metabolites and the potential functional relationships among metabolites, connected genes, and target diseases (Supplementary Figure 5).

## Discussion

Complex etiologies and atypical symptoms pose very large challenges to the early diagnosis of ASD. Urinary ^1^H-NMR analysis provides a fast and comprehensive assessment to detect potential biomarkers of ASD (28). Our study was based on the ^1^H-NMR analysis of urine samples from ASD and control groups to identify candidate metabolites and associated pathogenic mechanisms. Compared with other studies of urinary ^1^H-NMR metabolomics analysis of ASD, our study sample was large size, multi-center, and representative. Besides, our study used two normalization methods, creatinine normalization and total area normalization. The analysis using creatinine normalization showed no significant differences between the two groups, though the total area normalization did detect a difference. The variation of creatinine was confirmed by the total area normalization. Whiteley et al. also found that excretion of urinary creatinine in the group of pervasive developmental disorders, which included ASD, was significantly lower than controls (29). The abnormal creatinine metabolism might be caused by rigidity in food choice and various exclusion diets associated with ASD (30). Therefore, total area normalization was more suitable for our study.

The results obtained from the ^1^H-NMR analysis revealed that the levels of glycine, guanidinoacetic acid, creatine, hydroxyphenylacetylglycine, phenylacetylglycine and formate were higher in the ASD group than those in the control group. Moreover, the levels of 3-aminoisobutanoic acid, alanine, taurine, creatinine, hypoxanthine and N-methylnicotinamide were lower in the ASD group than those in the control group. The normalized peak areas of 3-aminoisobutanoic acid, creatine, creatinine, hypoxanthine, formate and N-methylnicotinamide differed significantly between the two groups. The metabolite levels of glycine, taurine, creatine and creatinine were consistent with those from previous reports (19, 31). Our study is the first to detect variations in the metabolites of hydroxyphenylacetylglycine in urine samples from children with ASD. The results of ROC analysis indicated that creatine, creatinine and hypoxanthine have the potential to be biomarkers for the diagnosis of ASD. The creatine/creatinine ratio slightly improved the diagnostic accuracy. Recent research has reported significant female-related alterations of creatine and creatinine, and the creatinine/creatine ratio might be a good predictor of ASD in female subjects (32). We further found that combining the metabolites creatine, creatinine and hypoxanthine as a potential diagnostic indicator can largely improve the diagnostic accuracy for ASD.

The extension study of the metabolic pathway analysis demonstrated a possible imbalance of amino acid metabolism in ASD children. Differential metabolites between the ASD and control groups involved glycine, serine and threonine metabolism, arginine and proline metabolism, taurine and hypotaurine metabolism, and glutathione metabolism pathways (Supplementary Figure 6). In accordance with previous observations, amino acid metabolism disorder plays an important role in the pathogenesis mechanism of ASD (33–37). Creatine and creatinine, which show significant metabolism alterations in ASD, play an essential role in maintaining a high level of energy supply for the brain (38). Studies have indicated that creatine deficiency occurs in some ASD cases, and creatine may be a therapeutic target for ASD (38–41). Abnormalities in creatinine might closely linked to abnormalities in creatine. Creatine is biosynthesized from glycine and arginine with an intermediate metabolite of guanidinoacetic acid. Glycine acts as an excitatory neurotransmitter in the early developmental stage. As the nervous system matures, it transforms to the major inhibitory neurotransmitter. If the transformation does not occur, an abnormal level of glycine may result in neural disorders, including ASD (42–44). The taurine and hypotaurine metabolism pathway also differed significantly between the two groups. Oxidative stress imbalance is considered to be important components of the pathophysiology of ASD, and antioxidant therapy may improve the prognosis of ASD (45, 46). Park et al. reported that taurine, as an antioxidant and regulator of inflammation, might be a valid biomarker for ASD (47). Combined with vitamin D3, taurine showed benefits in the treatment of ASD (48, 49). All metabolic pathways interact with each other and constitute a complex network with related genes and diseases (Supplementary Figure 5).

Many studies have reported that ASD is associated with abnormal gut microbial metabolism (50–52). Gut microbiota metabolites, including phenylalanine, tyrosine, hippurate and tryptophan, have been reported to be factors in the development of ASD (53–56). In our study, phenylacetylglycine, a gut microbial co-metabolite, had a slight variation between the two groups. Phenylacetylglycine is the end product of the phenylalanine metabolism pathway (57). However, there are no studies on the relationship between phenylacetylglycine and the pathogenesis of ASD.

## Limitations

There are some limitations in our study. At the cellular level, biochemical processes such as oxidative phosphorylation, redox reaction, and oxidative stress are regulated by circadian rhythms (58). About 50% of metabolites are thought to be rhythmic, involving metabolic pathways such as nucleotides, energy, oxidation, and carbohydrates (59). Studies indicated that disrupted circadian rhythm is closely related to ASD (60–62). Thus, in our study, inconsistent urine collection times may have affected the quality of metabolic analyses due to the restriction of research centers. A few small-scale sample studies have set healthy sibling group as control to remove the effects of confounding factors, such as heredity and environment (17, 19). However, our study lacks of healthy siblings as controls.

Though ^1^H-NMR analysis is the regular NMR method, some studies have used 2D HSQC-NMR to improve urinary screening in ASD. Compared to ^1^H-NMR analysis, ^1^H-^13^C HSQC-NMR analysis shows the advantage of improving the metabolite detection accuracy and the discrimination ability (18, 31). Moreover, it is necessary to compare metabolite levels that vary with ASD severity to better clarify the pathogenesis of ASD.

## Conclusions

In our study, ^1^H-NMR metabolomic analysis was used to investigate urinary metabolism patterns in the ASD and control groups. We revealed that urinary amino acid metabolites were significantly altered in children with ASD. A series of variations in amino acid metabolism pathways, including glycine, serine and threonine metabolism, arginine and proline metabolism, and taurine and hypotaurine metabolism might play important roles in the pathogenic mechanisms of ASD. Further studies of differential metabolites are needed to improve the understanding of ASD pathogenesis.

## Data Availability Statement

The raw data supporting the conclusions of this article will be made available by the authors, without undue reservation.

## Ethics Statement

The studies involving human participants were reviewed and approved by the institutional ethics committee at the Children's Hospital of Fudan University. Written informed consent to participate in this study was provided by the participants' legal guardian/next of kin.

## Author Contributions

YW conceived of the study. YM and HZ contributed to the analysis, synthesis and interpretation of the results, and wrote the manuscript. CL, LZ, and TW contributed to the sample collection. XZ, XL, LW, TL, XC, MM, YH, and EL contributed to the diagnosis of ASD and sample collection from each research center. YA, XX, WY, and YJ provided guidance for the study. All of the authors contributed to the preparation of the manuscript.

## Conflict of Interest

The authors declare that the research was conducted in the absence of any commercial or financial relationships that could be construed as a potential conflict of interest.

## Supplementary Material

Supplementary Figure 1 Urine sample preparation process.

Supplementary Figure 2 ROC curve of the metabolites. AUC, area under the ROC curve; SEN, sensitivity; SPE, specificity.

Supplementary Figure 3 ROC curve of the metabolites with relatively significant diagnostic values.

Supplementary Figure 4 Metabolic pathways associated with the differential metabolites.

Supplementary Figure 5 Metabolite-gene-disease interaction network.

Supplementary Figure 6 Differential metabolites associated with amino acid metabolism disorders.

Supplementary Table 1 Main metabolite attributions in the ^1^H-NMR spectra of urine samples.

Supplementary Table 2 Sex stratification analysis of the AUC of differential metabolites.

Supplementary Table 3 Age stratification analysis of the AUC of differential metabolites.

## Abbreviations

ASD: Autism spectrum disorder
PCA: Principal component analysis
OPLS-DA: Orthogonal projection to latent structure discriminant analysis
NMR: Nuclear magnetic resonance spectroscopy
DSM-5: Statistical manual of mental disorders 5
ADOS: Autism diagnostic observation schedule
ADI-R: Autism diagnostic interview-revised
SW: Spectral width
RD: Recycle delay
FID: Free induction decay
CV: Cross-validation
CV-ANOA: Variance analysis of the cross-validated residuals
ROC: Receiver operating characteristics
AUC: Area under the curve
FNR: False-negative rate
FPR: False-positive rate
PPV: Positive predictive value
NPV: Negative predictive value
LR+: Likelihood ratio positive
LR–: Likelihood ratio negative
MSEA: Metabolite sets enrichment analysis.
