# Supplementary figures and images for: Differential Metabolites in Chinese Autistic Children: A Multi-Center Study Based on Urinary 1H-NMR Metabolomics Analysis
Source: Front Psychiatry. 2021 May 11;12:624767. doi: 10.3389/fpsyt.2021.624767 (PMC8144639; doi:10.3389/fpsyt.2021.624767)

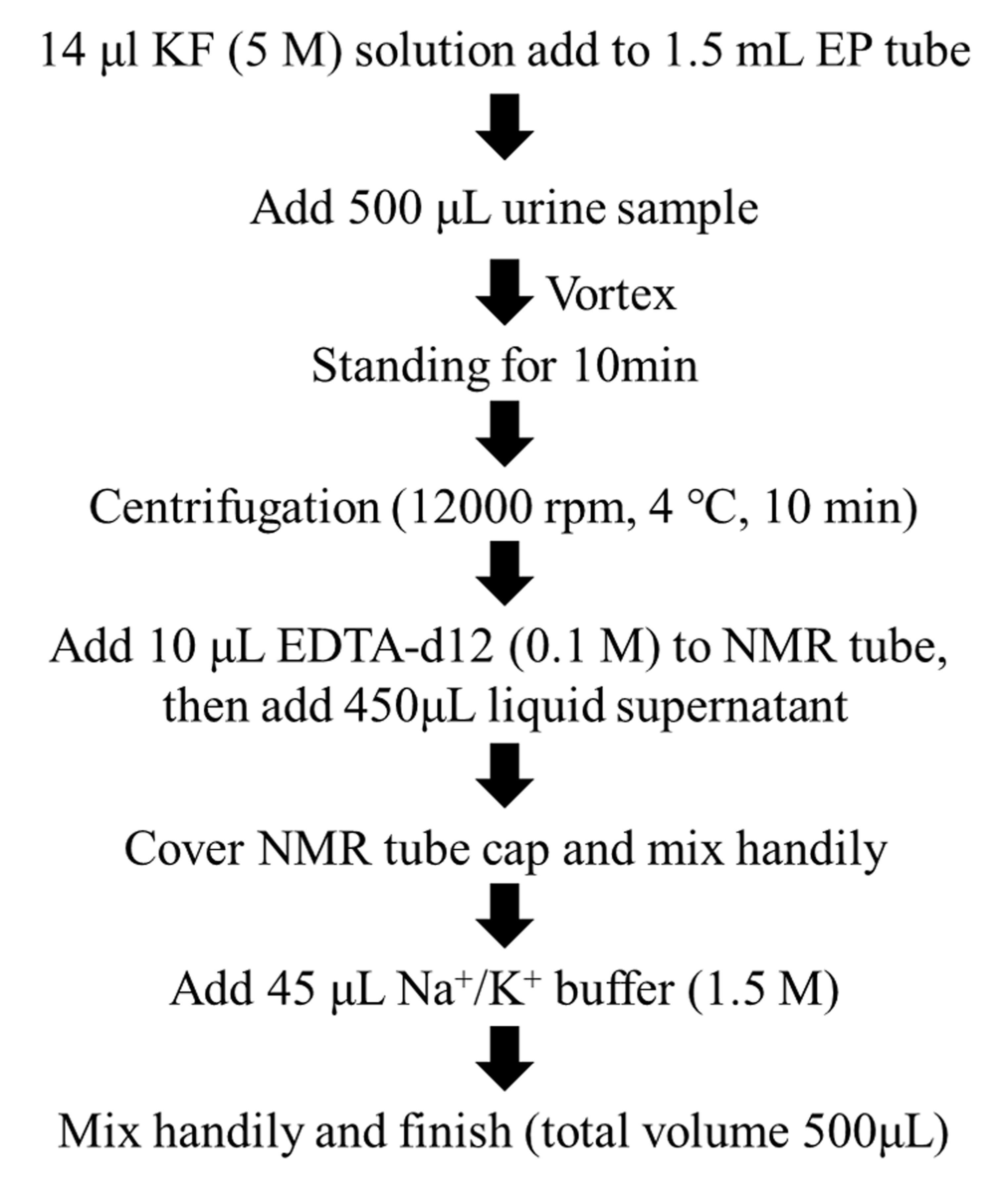

Supplement: Supplementary Figure 1 — Urine sample preparation process. [file Image_1.TIF]

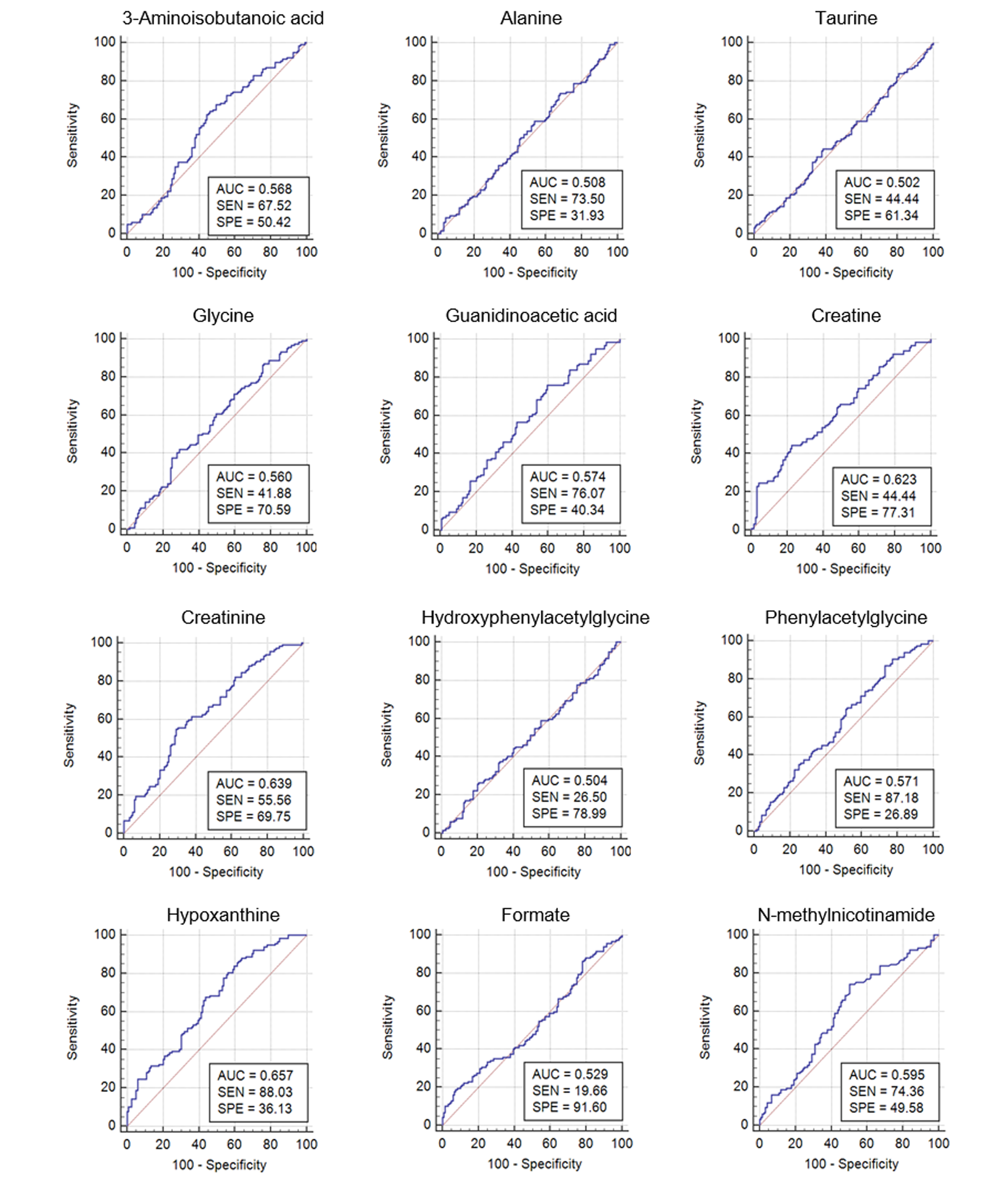

Supplement: Supplementary Figure 2 — ROC curve of the metabolites. AUC, area under the ROC curve; SEN, sensitivity; SPE, specificity. [file Image_2.TIF]

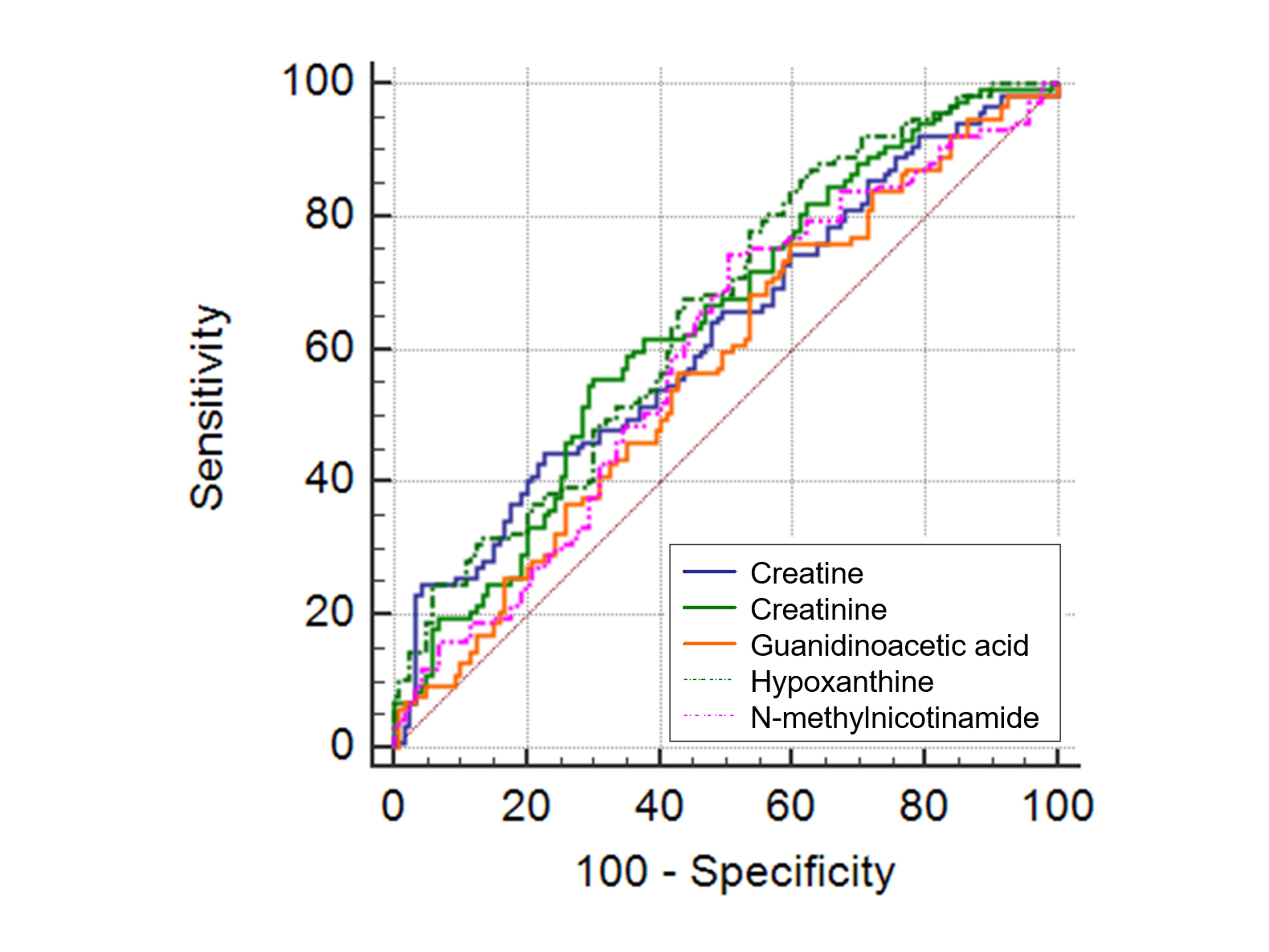

Supplement: Supplementary Figure 3 — ROC curve of the metabolites with relatively significant diagnostic values. [file Image_3.TIF]

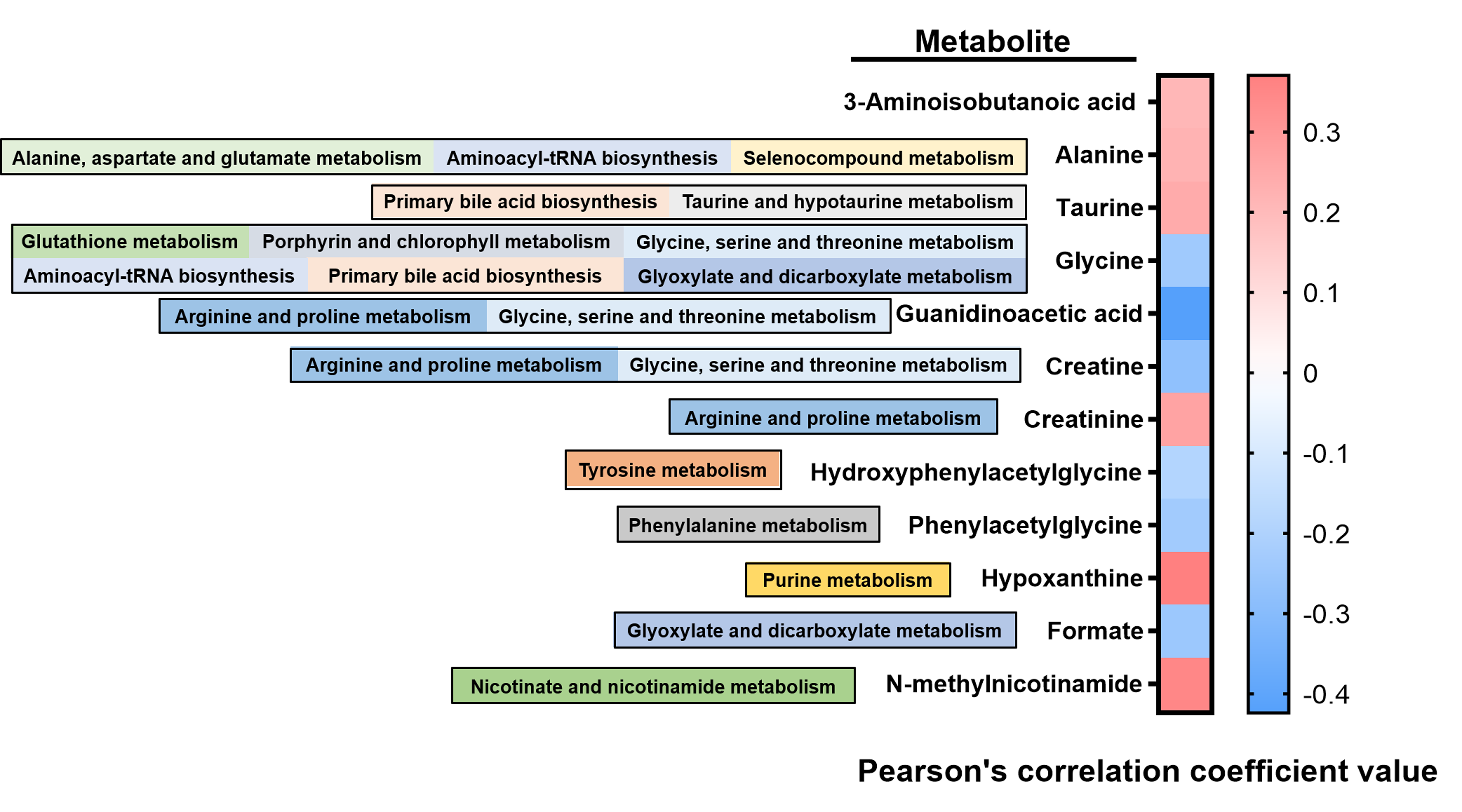

Supplement: Supplementary Figure 4 — Metabolic pathways associated with the differential metabolites. [file Image_4.TIF]

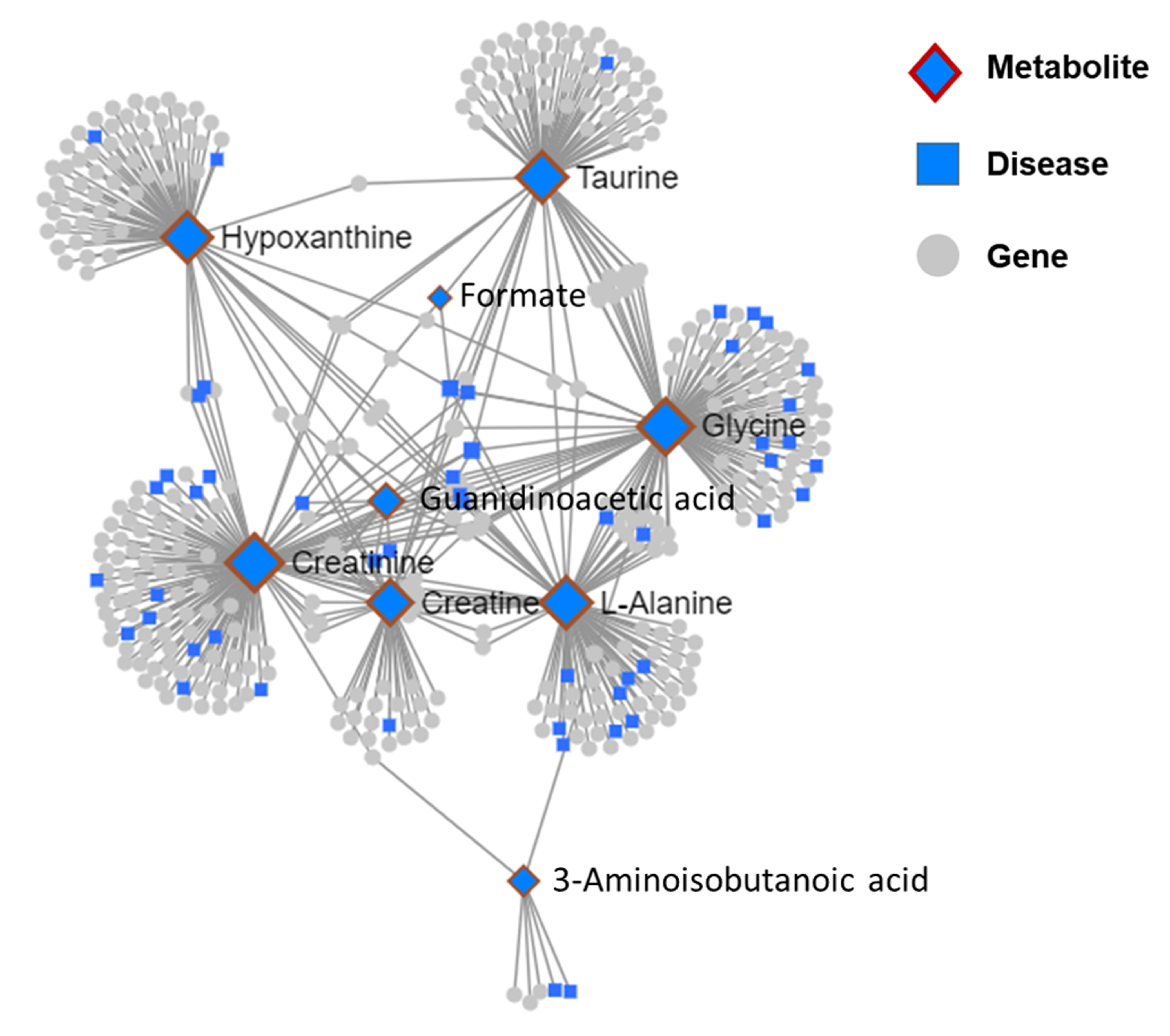

Supplement: Supplementary Figure 5 — Metabolite-gene-disease interaction network. [file Image_5.TIF]

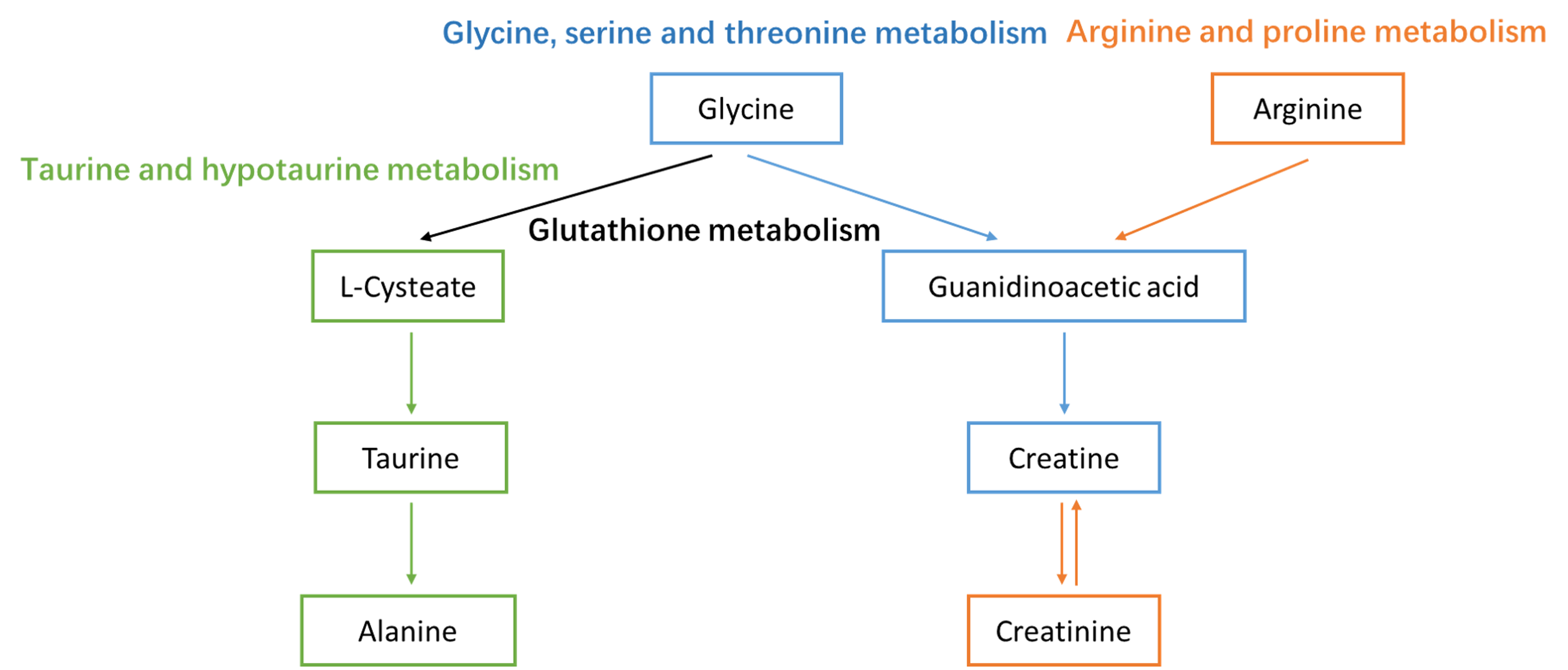

Supplement: Supplementary Figure 6 — Differential metabolites associated with amino acid metabolism disorders. [file Image_6.TIF]
