# Supplementary material for: Differential Metabolites in Chinese Autistic Children: A Multi-Center Study Based on Urinary 1H-NMR Metabolomics Analysis
Source: Front Psychiatry. 2021 May 11;12:624767. doi: 10.3389/fpsyt.2021.624767 (PMC8144639; doi:10.3389/fpsyt.2021.624767)
Supplement: Supplementary Table 1 — Main metabolite attributions in the 1H-NMR spectra of urine samples. [file Data_Sheet_1.docx]

**Additional file 1**

^1^H NMR spectra of urine samples were collected from ASD and control group. ^1^H-NMR spectra were transformed to chemical signal which correspond to metabolites. The attributions of main metabolites were listed in Table S1.

**Table S1 Main metabolite attributions in** **^1^H-NMR spectra of Urine Samples**

| NO. | Metabolites | Moieties | δ 1H (ppm) and multiplicity* |
| --- | --- | --- | --- |
| 1 | 3-Aminoisobutanoic acid | CH_3_ | 1.19(d) |
|  |  | αCH | 2.615(m) |
|  |  | βCH_2_ | 3.02(m) |
|  |  | βCH_2_' | 3.08(m) |
|  |  | COOH |  |
| 2 | Alanine | αCH | 3.78(q) |
|  |  | βCH_3_ | 1.48(d) |
| 3 | Creatine | CH_3_ | 3.04(s) |
|  |  | CH_2_ | 3.94(s) |
|  |  | C=NH |  |
|  |  | COOH |  |
| 4 | Creatinine | CH_3_ | 3.05(s) |
|  |  | CH_2_ | 4.06(s) |
|  |  | C=NH |  |
|  |  | C=O |  |
| 5 | Taurine | CH_2_SO_3_ | 3.28(t) |
|  |  | CH_2_NH_2_ | 3.43(t) |
| 6 | Glycine | CH_2_ | 3.57(s) |
|  |  | COOH |  |
| 7 | Guanidinoacetic acid | CH_2_ | 3.80(s) |
| 8 | Hydroxyphenylacetylglycine | CH_2_ | 3.85(s) |
|  |  | CH_2_ | 3.83(s) |
|  |  | 2 or 6-CH | 6.87(d) |
|  |  | 3 or 5-CH | 7.16(m) |
|  |  | COOH |  |
| 9 | Phenylacetylglycine | 3 or 5-CH | 7.43(m) |
|  |  | 2 or 6-CH | 7.37(m) |
|  |  | 4-CH | 7.37(m) |
|  |  | CH_2_ | 3.66(s) |
|  |  | C=O |  |
| 10 | Hypoxanthine | 8-CH | 8.20(s) |
|  |  | 2-CH | 8.22(s) |
| 11 | Formate | CH | 8.46(s) |
|  |  | COOH |  |
| 12 | N-methylnicotinamide | 2-CH | 9.29(s) |
|  |  | 4-CH | 8.91(d) |
|  |  | 6-CH | 8.97(d) |
|  |  | CH_3_ | 4.48(s) |
| 13 | Lactate | αCH | 4.12(q) |
|  |  | βCH_3_ | 1.33(d) |
|  |  | COOH |  |
| 14 | Acetate | CH_3_ | 1.93(s) |
|  |  | COOH |  |
| 15 | Succinate | CH_2_ | 2.41(s) |
|  |  | COOH |  |
| 16 | Citrate | CH_2_ | 2.53(d) |
|  |  | CH_2_' | 2.67(d) |
|  |  | C-OH |  |
|  |  | COOH |  |
| 17 | Urea | NH_2_ | 5.82(bs) |
| 18 | Fumarate | CH | 6.52(s) |
|  |  | COOH |  |
| 19 | 2-Hydroxyisobutyrate | CH_3_ | 1.36(s) |
| 20 | Dimethylamine (DMA) | CH_3_ | 2.72(s) |
| 21 | Cis-Aconitate | CH | 5.71(s) |
|  |  | CH_2_ | 3.12(s) |
|  |  | COOH |  |
| 22 | Hippurate | 3 or 5-CH | 7.55(dd) |
|  |  | 4-CH | 7.64(t) |
|  |  | 2 or 6-CH | 7.84(dd) |
|  |  | αCH_2_ | 3.97(s) |
|  |  | NH | 8.56(s) |
|  |  | COOH |  |
| 23 | Acetamide | CH_3_ | 1.99(s) |
|  |  | C=O |  |
| 24 | Tryptamine | 4-CH | 7.72(dd) |
|  |  | 5-CH | 7.20(td) |
|  |  | 7-CH | 7.53(td) |
|  |  | 6-CH | 7.28(td) |
| 25 | 4PY(N1-methyl-4-pyridone-3-carboxamide) | 3-CH | 6.70(d) |
|  |  | 2-CH | 7.82(d) |
|  |  | 6-CH | 8.56(d) |
|  |  | N-CH_3_ | 3.90(s) |
| 26 | 2PY | 3-CH | 6.67(d) |
|  |  | 4-CH | 7.98(d) |
|  |  | 6-CH | 8.34(d) |
|  |  | N-CH_3_ | 3.97(s) |
| 27 | Guanine | CH | 7.69(s) |
|  |  | C=C |  |
|  |  | C=C |  |
|  |  | C-NH_2_ |  |
| 28 | Trimethylamine (TMA) | CH_3_ | 2.87(s) |
| 29 | Trigonelline | 1-CH | 9.13(s) |
|  |  | 2 or 4-CH | 8.85(dd) |
|  |  | 3-CH | 8.05(d) |
|  |  | CH_3_ | 4.45(s) |
| 30 | Glycoprotein | NHCOCH_3_ | 2.03(s) |
| 31 | Trimethylamine N-oxide (TMAO) | CH_3_ | 3.27(s) |
| 32 | Uracil | 5-CH | 5.81(d) |
|  |  | 6-CH | 7.53(d) |
| 33 | 3-Hydroxyisobutyric acid | CH_3_ | 1.07(d) |
|  |  | CH | 2.5(m) |
|  |  | CH_2_ | 3.55(m) |
|  |  | CH_2_' | 3.70(m) |
| 34 | Valine | αCH | 3.62(d) |
|  |  | βCH | 2.27(m) |
|  |  | γCH_3_ | 1.05(d) |
|  |  | γ′CH_3_ | 0.99(d) |
|  |  | COOH |  |
| 35 | 3-Hydroxyisovalerate | CH_3_ | 1.27(s) |
|  |  | CH_2_ | 2.38(s) |
|  |  | C |  |
| 36 | Succinimide | CH_2_ | 2.78(s) |
|  |  |  |  |
| 37 | Methylguanidine | CH_3_ | 2.83(s) |
|  |  | C=O |  |
| 38 | Histidine | 4-CH | 7.15(s) |
|  |  | 2-CH | 8.04(s) |
| 39 | Salicyluric acid | 3-CH | 7.78(dd) |
|  |  | 4-CH | 6.99(dd) |
|  |  | CH_2_ | 3.95(s) |
|  | Unknown | CHx | 1.145(d) |
|  |  | CHx | 3.723(m) |
| *s, singlet; d, doublet; t, triplet; q, quartet; m, multiplet; dd, doublet of doublet; bs, broad signlet. | | | |
|  |  |  |  |

Results of AUC analysis stratified by gender and age were showed in Table S2 and Table S3. There was no significant difference in AUC of metabolite between male and female. Compared with age stratification of 7-9 years old, metabolites of guanidinoacetic acid and creatine showed significantly higher diagnostic accuracy for ASD in the age stratification of 13-15 years old (AUC of guanidinoacetic = 0.802, 95% CI 0.566 to 0.944, *P* = 0.0282. AUC of creatine = 0.823, 95% CI 0.589 to 0.955, *P* = 0.0344).

**Table S2 Gender stratification analysis of AUC of differential metabolites**

| Metabolites | Male | | Female | | *P* value |
| --- | --- | --- | --- | --- | --- |
|  | AUC | 95% CI | AUC | 95% CI |  |
| 3-Aminoisobutanoic acid | 0.591 | 0.515-0.665 | 0.579 | 0.419-0.728 | 0.9057 |
| Alanine | 0.518 | 0.442-0.594 | 0.505 | 0.348-0.660 | 0.8925 |
| Taurine | 0.521 | 0.445-0.597 | 0.624 | 0.464-0.767 | 0.2953 |
| Glycine | 0.585 | 0.509-0.659 | 0.502 | 0.346-0.658 | 0.4151 |
| Guanidinoacetic acid | 0.585 | 0.508-0.659 | 0.548 | 0.389-0.700 | 0.7054 |
| Creatine | 0.653 | 0.577-0.723 | 0.516 | 0.359-0.671 | 0.2066 |
| Creatinine | 0.671 | 0.596-0.740 | 0.502 | 0.346-0.658 | 0.0852 |
| Hydroxyphenylacetylglycine | 0.522 | 0.445-0.597 | 0.645 | 0.484-0.785 | 0.2155 |
| Phenylacetylglycine | 0.549 | 0.472-0.624 | 0.606 | 0.446-0.752 | 0.5625 |
| Hypoxanthine | 0.654 | 0.579-0.724 | 0.740 | 0.584-0.861 | 0.3284 |
| Formate | 0.541 | 0.464-0.616 | 0.509 | 0.352-0.665 | 0.7584 |
| N-methylnicotinamide | 0.581 | 0.504-0.655 | 0.679 | 0.519-0.813 | 0.3032 |

**Table S3 Age stratification analysis of AUC of differential metabolites**

| Metabolites | 7-9 years | | 10-12 years | | 13-15 years | | *P* value | | |
| --- | --- | --- | --- | --- | --- | --- | --- | --- | --- |
|  | AUC | 95% CI | AUC | 95% CI | AUC | 95% CI | 7-9 y  vs  10-12 y | 7-9 y  vs  13-15 y | 10-12 y  vs  13-15 y |
| 3-Aminoisobutanoic acid | 0.534 | 0.418-0.647 | 0.652 | 0.558-0.739 | 0.646 | 0.404-0.843 | 0.1587 | 0.4558 | 0.9632 |
| Alanine | 0.529 | 0.413-0.642 | 0.517 | 0.421-0.611 | 0.615 | 0.374-0.820 | 0.8929 | 0.5646 | 0.4976 |
| Taurine | 0.526 | 0.410-0.639 | 0.500 | 0.405-0.595 | 0.531 | 0.299-0.754 | 0.7679 | 0.9755 | 0.8531 |
| Glycine | 0.503 | 0.389-0.618 | 0.573 | 0.477-0.665 | 0.134 | 0.414-0.851 | 0.4183 | 0.3078 | 0.5649 |
| Guanidinoacetic acid | **0.501** | **0.386-0.615** | 0.576 | 0.480-0.668 | **0.802** | **0.566-0.944** | 0.3832 | **0.0282** | 0.0863 |
| Creatine | **0.561** | **0.444-0.672** | 0.632 | 0.536-0.720 | **0.823** | **0.589-0.955** | 0.4036 | **0.0344** | 0.1038 |
| Creatinine | 0.670 | 0.555-0.771 | 0.565 | 0.469-0.657 | 0.802 | 0.566-0.944 | 0.1978 | 0.3238 | 0.0710 |
| Hydroxyphenylacetylglycine | 0.555 | 0.438-0.666 | 0.538 | 0.442-0.632 | 0.729 | 0.487-0.900 | 0.8491 | 0.2036 | 0.1456 |
| Phenylacetylglycine | 0.527 | 0.412-0.641 | 0.629 | 0.534-0.718 | 0.510 | 0.281-0.737 | 0.2309 | 0.9177 | 0.4495 |
| Hypoxanthine | 0.701 | 0.587-0.799 | 0.630 | 0.535-0.719 | 0.646 | 0.404-0.843 | 0.3708 | 0.7038 | 0.9110 |
| Formate | 0.503 | 0.388-0.617 | 0.505 | 0.409-0.600 | 0.677 | 0.434-0.865 | 0.9820 | 0.2749 | 0.2640 |
| N-methylnicotinamide | 0.595 | 0.479-0.704 | 0.583 | 0.487-0.674 | 0.667 | 0.424-0.858 | 0.8856 | 0.6351 | 0.5664 |
